# Physical fighting among adolescents in eastern Ethiopia: a cross-sectional study

**DOI:** 10.1186/s12889-021-11766-w

**Published:** 2021-09-24

**Authors:** Agumasie Semahegn, Yadeta Dessie, Nega Assefa, Chelsey R. Canavan, Yemane Berhane, Wafaie W. Fawzi

**Affiliations:** 1 College of Health and Medical Sciences, School of Public Health, Haramaya University, P.O. Box 235, Harar, Ethiopia; 2 Department of Global Health and Population, Harvard T.H. Chan School of Public Health, 677 Huntington Avenue, Boston, MA 02115 USA; 3 Department of Epidemiology, Addis Continental Institute of Public Health, Ayat Zone 8, Addis Ababa, Ethiopia

**Keywords:** Violence, Physical attack, Physical fight, Adolescent, Ethiopia

## Abstract

**Background:**

Physical fights have been a common health problem among adolescents, and approximately a million adolescents’ lives are lost due to violence-related incidents worldwide. There is a lack of information on the burden of adolescents’ physical fights in eastern Ethiopia. Hence, the study aims to estimate the magnitude and assess factors associated with physical attacks and fighting among adolescents in eastern Ethiopia.

**Methods:**

A community-based cross-sectional study was conducted among 2424 adolescents in eastern Ethiopia in 2016. Simple random sampling was used to recruit study participants. Data were collected by trained interviewers using a structured questionnaire developed by the Africa Research, Implementation Science and Education (ARISE) network and adapted from the World Health Organization Global school-based student health survey. Descriptive statistics, binary and multivariable logistic regression were performed. Statistical associations were determined using adjusted odds ratio (AOR) at 95% Confidence Intervals (95% CIs) and *P*-value < 0.05.

**Results:**

Prevalence of physical attacks and physical fights was 5.8%, and 26.4%, respectively. Adolescents who attended school (AOR 0.4, 95% CI: 0.2–0.9) and who chewed Khat (AOR 0.4, 95% CI: 0.2–0.8) were less likely to experience physical attacks. Male adolescents were two times more likely to engage in physical fights than female adolescents (AOR 2.4, 95% CI: 1.8–3.2). In-school adolescents who attended secondary (AOR 0.4, 95% CI: 0.2–0.7) or tertiary level of education (AOR 0.2, 95% CI: 0.1–0.7) were less likely to participate in physical fighting than those with primary level education. Adolescents who had ever engaged in physical work to earn money for food or drink were 1.9 times more likely to be physically attacked compared to those who had not (AOR 1.9, 95% CI: 1.0–3.5).

**Conclusion:**

Physical attacks and fights were found to be common experiences of adolescents in eastern Ethiopia. Future research and programs should emphasize preventive health programs for reducing violence and promoting school enrolment and retention.

## Background

Adolescents (10–19 years) account for approximately 1.8 billion people, which is almost a quarter of the world’s population; adolescents account for about one-third of the population of sub-Saharan Africa [1]. In Ethiopia, adolescents comprise 24.8% of the total population [2]. Life experiences during the period of adolescence have implications for their health later in adulthood, and also lead to important effects on the economic development and social prospects of a country [1]. Investing in adolescent health and wellbeing may therefore benefit health in adulthood [2].

Violence (both physical fights and attacks) is a global public health concern that has a serious impact on adolescent growth and development, as well as cost implications for health care, social welfare, criminal justice services, and also costs related to productivity losses [3]. Violence is defined as the intentional use of threatened or actual physical force or power against oneself, another person, or a group or community, which results in or has a high likelihood of resulting in injury, death, psychological harm, poor development, or deprivation [4]. Violence causes nearly 1.4 million deaths per year, worldwide [5]. Many adolescents have died prematurely due to accidents, suicide, violence, and other preventable or treatable illnesses [6]. Globally, female adolescents face different forms of violence depending on their age and marital status. However, both female and male adolescents are subjected to physical and emotional abuse by those who have power and authority over them, including their peers [7].

Adolescent physical fighting is an important public health agenda, which contributes to the global burden of disease associated with injuries and illness as a consequence of physical fights and attacks, however, research evidence on young people’s well-being is very limited and remain as a neglected and yet pressing issue [8, 9]. In many parts of the world, particularly high income countries, injuries and self-harm are common causes of morbidity and mortality among adolescents [8]. Addressing preventable physical attacks, fighting, and other forms of violence will have tremendous socio-economic benefits [10]. Yet, in many low and middle income countries, including Ethiopia, research on violent behavior among adolescents is lacking to inform policy and programs. Therefore, the study aims to estimate the magnitude of physical attacks/violence, fighting, and assess associated factors among adolescents in eastern Ethiopia. This study provides insight into the levels of and associated factors for these conditions to inform health providers, program planners, researchers, and public health experts working on adolescent health in this region.

## Methods

### Setting and participants

A community-based cross-sectional study was conducted among adolescents in rural (Kersa) and urban (Harar) Health Demographic Surveillance System (HDSS) sites in eastern Ethiopia from February 1, 2016 to March 30, 2016. The HDSSs were established in 2007. The Harar HDSS encompasses six sub-districts including approximately 9000 households with 32,000 people undergoing periodic surveys. The Kersa HDSS is located in the Kersa district of the east Hararghe Zone in the Oromia Regional State. Details on the Kersa HDSS have been published elsewhere [11]. The study was conducted among adolescents (10–19 years of age) who lived in the study area. Adolescent study participants were randomly recruited from 18 sub-districts (six urban and 12 rural). Adolescents were excluded if they were not willing to participate in the study, seriously ill, or if their parents refused consent.

### Sample size determination and sampling

Sample size was calculated considering 95% significance level, 2% margin of error (d) and a proportion of 14% (p), then 5% for non-response and a design effect of 2, which yields 2424. A total of 18 sub-districts (Harar (urban) =6 and Kersa (rural) =12) were included in the survey. Households with an adolescent were randomly selected using simple random sampling method from the HDSS databases. When two or more adolescents were found within the selected household, each was considered for the interview. Sub-district name, village name, household identification, and household head names from the HDSS database were used to identify locations of selected households in the study area.

### Dependent variables’ operational definition

#### Physical attack

A physical attack was defined as an incident “when an adolescent is intentionally harmed by a person in the context of unbalanced strength and power” [12]. This variable was assessed using the question, “during the past 12 months, how many times were you physically attacked?” For participants who had not been physically attacked, the response was converted to a dichotomous variable, coded as “no incident = 0,” and for those who had been physically attacked at least one time, the response was coded as “yes = 1.”

#### Physical fight

A physical fight was defined as “when two or more adolescents of about the same strength and power choose to fight each other” [12]. This variable was assessed using the question, “during the past 12 months, how many times were you in a physical fight?” For participants who had not been in a physical fight, the response was converted to a dichotomous variable coded as “no incident = 0,” and for those participants who had been in at least one physical fight, the response was coded as “yes = 1.”

### Independent variables

#### Socio-demographic and behavioral variables

Age, sex, schooling (in-school or out-of-school), educational status (grade level), having worked to earn money for food/drink, alcohol consumption, and consumption of the psycho-stimulant khat plant.

#### Parental/guardian characteristics

Age, educational status, occupation, family size, and wealth index.

#### Wealth index

A Principal Component Analysis (PCA) was performed to quantify the wealth-index of households. Household assets (electricity, wall-clock, radio, black/white television, colored television, mobile phone, refrigerator, chest/deep freezer, electric generator/invertor, washing machine, computer, digital photo camera, non-digital photo camera, video deck, DVD/CD, sewing machine, bed, table, cabinet/cupboard, bicycle, motor bicycle or motor scooter, car or truck, boat with a motor, boat without a motor, and solar source of energy), main source of drinking water, and type and availability of toilet facilities were included in the PCA. Kaiser-Meyer-Olkin (KMO) and Bartlett’s test of sphericity tests were used for load and sample adequacy. Correlation-matrix, covariate-matrix and Eigenvalues were used to extract factors. Any anti-image correlation values less than 0.5 were excluded step-wisely. Eigenvalues greater than 60% were consider as an adequate sample. Finally, nine household assets (bed, DVD/CD, cabinet/cupboard, table, electricity, refrigerator, color television) were used to rank the wealth-index of adolescents’ households, which were then ranked in quintiles.

### Data collection techniques

Data were collected using a structured questionnaire developed by the Africa Research, Implementation Science and Education (ARISE) network and adapted from the WHO global school-based student health survey [13]. The data collection tool was comprised of comprehensive variables for assessing adolescent health, including: socio-demographic and socio-economic characteristics, family relationships, substance use (alcohol and khat), physical attack/violence, and physical fighting. Pretesting was conducted among 5% (*n* = 120) of the total sample size (*n* = 2424). Amendments were made to the content (khat is contextualized among the psycho-stimulant substances), language and format of the questionnaire to improve cultural appropriateness and clarity. Adolescents were interviewed face-to-face by a data collector of the same sex. Data collectors were carefully selected, trained and closely supervised on a daily basis. For this purpose, a minimum of bachelor’s degree in public health or nursing, as well as prior experience in the supervision of field work, were required to be selected as a data collector. Different corrective measures were taken by supervisors and investigators team as deemed necessary. Eventually, participants in the pretest were excluded from the main study. Training and supportive supervision were provided to data collectors.

### Statistical methods

Electronic and paper records were stored at Haramaya University College of Health and Medical Sciences. A standard coding guide, data entry guide, and detailed computer editing specifications were prepared by the research team. Data were double-entered to EpiData (version 3.0.2) software. Then, the data were exported from EpiData to statistical package for the social sciences (SPSS) (version 23.0) for analysis. Descriptive statistics (frequency, percentage, mean and standard deviations) were calculated. Data were analyzed using logistic regression to determine the relationships between the dependent and independent variables. Models were assessed using the Hosmer-Lemeshow fitness test. A total of 16 independent variables (sex, age, residency, currently in-school, school grade level for in-school adolescents, school grade completed for out-of-school adolescents, engaged in work for food or drink, family size, parents age, occupation, wealth index, khat chewing, alcohol use, and having physical education class in the school year) were considered in the binary logistic regression. Factors with a *p*-value less than 0.25 in the bivariate analysis were included in multivariable models. Finally, adjusted odds ratio (AOR) with 95% CI at *p* value less than 0.05 was used to declare an association between independent and dependent variables as statistically significant.

## Results

The study participants’ response rate was 83% (2010/2424). The mean age was 13. 8 years (±2.7). Sixty-one percent of study participants were in early adolescence (10–14 years old), 51.1% were female, and 76.7% attended school during the survey period. In addition, 41.6% were currently in primary school (grades 1–4) and 52.7% lived in an urban area. Almost one-in-five (18.4%) adolescents engaged in physical work to earn money for food and or drink (Table 1). The mean family household consisted of 5.8 (±2.1) members. The majority of households (70.1%) had more than four household members, and 74.2% of adolescents were living with both parents. Male and female parents/guardians who did not have a formal education were 45 and 51.7%, respectively. The mean age of male and female parents/guardians was 56.1 years (±22.8) and 46.2 years (±21.1), respectively (Table 2).

**Table 1:** Socio-demographic characteristics of the adolescents in eastern Ethiopia (*n* = 2010)

Variable	Categories	n (%)
Adolescent age (years)	10–14	1229 (61.1)
	15–17	543 (27.0)
	18–19	238 (11.8)
Sex	Male	982 (48.9)
	Female	1028 (51.1)
School enrolment	Yes	1541 (76.7)
	No	469 (23.3)
Residency	Urban (Harar)	1059 (52.7)
	Rural (Kersa)	951 (47.3)
In-school grade level	Primary (1–4)	641 (41.6)
	Primary (5–8)	527 (34.2)
	Secondary (9–12)	327 (21.2)
	Tertiary (college level)	46 (3.0)
Out-of-school grade level	None	285 (60.8)
	Primary (1–4)	113 (24.1)
	Primary (5–8)	51 (10.9)
	Secondary (9–12)	20 (4.3)
	Tertiary (college levels)	0 (0.0)
Engaged in physical work for food/drink	Yes	370 (18.4)
	No	1640 (81.6)
Have you consumed Khat in your lifetime?	Yes	326 (16.2)
	No	1684 (83.8)
Alcohol	yes	39 (26.4)
	No	109 (73.6)

**Table 2:** Socio-demographic characteristics of adolescent’s parents/guardians in eastern Ethiopia (*n* = 2010)

Variables	Categories	n (%)
**Parent/guardian status**	Both alive	1707 (84.9)
	Mother alive	200 (10.0)
	Father alive	58 (2.9)
	Both not alive	34 (1.7)
	Do not know	11 (0.5)
**Currently living with**	Both parents	1491 (74.2)
	Mother alone	283 (14.1)
	Father alone	53 (1.6)
	Male guardian alone	38 (1.9)
	Female guardian alone	90 (4.5)
	By myself	20 (1.0)
	Others specify	35 (1.7)
**Father/male guardian educational status**	None	654 (45.0)
	Primary (1–8)	333 (22.9)
	Secondary (9–12)	309 (21.3)
	Tertiary (college level)	156 (10.7)
**Father/male guardian occupation**	Farmer	768 (48.2)
	Merchant	185 (11.6)
	teacher	17 (1.1)
	Government employee	259 (16.2)
	Others	366 (22.9)
**Age of father/male guardian (years)**	Mean age	56.1(±22.8)
	25–34	55 (3.7)
	35–44	513 (34.3)
	45–64	578 (38.7)
	65+	349 (23.3)
**Educational status of mother/female guardian**	None	787 (51.7)
	Primary (1–8)	354 (23.2)
	Secondary (9–12)	282 (18.5)
	Tertiary	100 (6.6)
**Occupation of mother/female guardian**	Farmer	349 (33.1)
	Merchant	309 (18.6)
	Teacher	14 (0.8)
	Government employee	169 (10.2)
	Others	617 (37.2)
**Age of mother/female guardian (years)**	Mean age	46.2(±21.1)
	Less than 25	22 (1.3)
	25–34	349 (21.0)
	35–44	757 (45.7)
	45–64	296 (71.9)
	65+	234 (14.1)
**Household family member/size\**	Mean family size	5.8 (±2.1)
	≤ 4	601 (29.9)
	> 4	1409 (70.1)
**Wealth index**	Lowest	657 (32.7)
	Second	265 (13.2)
	Middle	274 (13.6)
	Fourth	409 (20.3)
	Highest	405 (20.1)
**Hunger during last 30 days**	Never	1452 (72.2)
	Rarely	281 (14.0)
	Sometimes	246 (12.2)
	Usually	29 (1.4)
	Always	2 (0.1)

### Physical attack

During the 12 months preceding this survey, 5.8% of adolescents had been physically attacked. Of these adolescents, 4.7% had been attacked once, and the remaining 1.1% had been attacked multiple times. The experience of being physically attacked was higher among female adolescents than male adolescents (Table 3).

**Table 3:** Factors associated with adolescents being physically attacked in eastern Ethiopia (*n* = 2010)

Variable	Category	Physically attacked		COR(95%CI)-	AOR (95%CI)
		Yes, n (%)	No, n (%)		
**Adolescent age (years)**	10–14	72 (5.9)	1157 (94.1)	1.3 (0.7–2.5)	2.2 (0.5–9.9)
	15–17	34 (6.3)	509 (93.7)	1.2 (0.7–2.8)	2.3 (0.6–9.9)
	18–19	11 (4.6)	227 (95.4)	1.0	1.0
**Sex**	Male	44 (4.5))	938 (95.5)	1.6 (1.1–2.4)**	1.5 (0.8–2.6)
	Female	73 (7.1)	955 (92.9)	1.0	1.0
**Residency**	Urban (Harar)	46 (4.3)	1013 (95.7)	1.0	1.0
	Rural (Kersa)	71 (7.5)	880 (92.5)	1.8 (1.2–2.6)**	1.4 (0.4–5.3)
**Currently enrolled in school**	Yes	81 (5.3)	1460 (94.7)	0.7 (0.4–1.0)	0.4 (0.2–0.9)**
	No	36 (7.7)	433 (92.3)	1.0	1.0
**In-school grade level**	Primary (1–4)	45 (7.0)	596 (93.0)	1.0	1.0
	Primary (5–8)	26 (4.9)	501 (95.1)	0.7 (0.4–1.1)	0.7 (0.4–1.4)
	Secondary (9–12)	8 (2.4)	319 (97.6)	0.3 (0.2–0.7)	0.4 (0.2–1.1)
	Tertiary (college level)	2 (4.3)	44 (95.7)	0.6 (0.1–2.6)**	1.1 (0.2–5.2)
**Engaged in physical work for food /drink**	Yes	36 (9.7)	334 (90.3)	2.1 (1.4–3.1)**	1.9 (1.0–3.5)
	No	81 (4.9)	1559 (95.1)	1.0	1.0
**Household members size**	≤ 4 members	48 (5.1)	900 (94.9)	1.0	1.0
	> 4 members	69 (6.5)	993 (93.5)	1.3 (0.9–1.9)	1.1 (0.6–2.1)
**Father/male guardian age (years)**	25–34	2 (3.6)	53 (96.4)	0.4 (0.1–1.7)	0.7 (0.1–3.1)
	35–44	25 (4.9)	488 (95.1)	0.6 (0.3–0.9)	0.7 (0.4–1.5)
	45–64	30 (5.2)	548 (94.8)	0.6 (0.4–1.0)	0.7 (0.3–1.4)
	65+	30 (8.6)	319 (91.4)	1.0	1.0
**Wealth index**	Lowest	45 (6.8)	612 (93.2)	1.0	1.0
	Second	19 (7.2)	246 (92.8)	1.1 (0.6–1.8)	1.1 (0.5–2.5)
	Middle	17 (6.2)	257 (93.8)	0.9 (0.5–1.6)	1.7 (0.5–5.7)
	Fourth	22 (5.4)	387 (94.6)	0.8 (0.5–1.3)	1.7 (0.4–7.3)
	Highest	14 (3.5)	391 (96.5)	0.5 (0.3–0.9)	0.8 (0.2–3.6)
**Khat chewing**	Yes	84 (5.0)	1600 (95.0)	0.5 (0.3–0.7)**	0.4 (0.2–0.8)**
	No	33 (10.1)	293 (89.9)	1.0	1.0

**p < 0.05: significant association, *COR* Crude Odds Ratio, *AOR* Adjusted Odds Ratio

Ten independent variables with *p*-values ≤ 0.25 from the binary logistic regression were included in multiple logistic regression model. These independent variables were: adolescent age, sex, residence, currently in-school, school grade level for in-school adolescents, engaged in work for food or drinks, family size, parents’ age, wealth-index and adolescent khat-chewing. Two independent variables were found to be significantly associated with adolescents’ being physically attacked in the final logistic regression model. Adolescents who were attending school during the survey were 59% less likely to be physically attacked than adolescents who were not attending school (AOR 0.4, 95% CI 0.2–0.9). Adolescents who had ever chewed khat were 58% less likely to be engaged in physical attacks than their counterparts (AOR 0.4, 95% CI 0.2–0.8) (Table 3).

### Physical fights

Physical fighting was reported by 26.4% of adolescents. Of these participants, 12.0% had a one-time physical fight, and 14.4% reported that they had experienced two physical fights. In multivariable analyses, some predictors were found to be significantly associated adolescents’ physical fights. Of these, male adolescents were 2.4 times more likely to be involved in a physical fight than female adolescents (AOR 2.4, 95% CI 1.8–3.2). The odds of being involved in physical fights among in-school adolescents who had attended secondary school were 62% less likely (AOR 0.4, 95% CI 0.2–0.7), and tertiary level adolescents were 80% less likely (AOR 0.2, 95% CI: 0.1–0.7) than primary level adolescents (grades 1–4) (Table 4).

**Table 4:** Factors associated with adolescent physical fighting in eastern Ethiopia (*n* = 2010)

Variable	categories	Physical-fighting		COR (95% CI)	AOR(95% CI)
		Yes n(%)	No n(%)		
**Adolescent age (years)**	10–14	372 (30.3)	857 (69.7)	1.9 (1.3–2.6)**	1.4 (0.8–2.5)
	15–17	113 (20.8)	430 (79.2)	1.1 (0.8–1.7)	1.1 (0.6–1.9)
	18–19	45 (18.9)	193 (81.1)	1.0	1.0
**Sex**	Male	188 (19.1)	794 (80.9)	2.1 (1.7–2.6)**	2.4 (1.8–3.2)**
	Female	342 (33.3)	686 (66.7)	1.00	1.00
**Residency**	Urban	290 (27.4)	769 (72.6)	1.1 (0.9–1.4)	1.8 (0.8–4.1)
	Rural	240 (25.2)	711 (74.8)	1.0	1.0
**Currently in-school**	Yes	414 (26.9)	1127 (73.1)	1.0	1.0
	No	116 (24.7)	353 (75.3)	0.9 (0.7–1.1)	1.0 (0.8–1.1)
**In-school grade level**	Primary (1–4)	199 (31.0)	442 (69.0)	1.0	1.0
	Primary (5–8)	159 (30.2)	368 (69.8)	1.0 (0.8–1.2)	0.8 (0.6–1.1)
	Secondary (9–12)	52 (15.9)	275 (84.1)	0.4 (0.3–0.6)**	0.4 (0.2–0.7)**
	Tertiary (college level)	4 (8.7)	42 (91.3)	0.2 (0.1–0.6)**	0.2 (0.1–0.7)**
**Engaged in physical work for food /drink**	Yes	98 (26.5)	272 (73.5)	1.0 (0.8–1.3)	0.9 (0.6–1.3)
	No	1208 (73.7)	432 (26.3)	1.0	1.0
**Mother/female guardian education**	None	208 (26.4)	579 (73.6)	1.0	1.0
	Primary (1–8)	106 (29.9)	248 (70.1)	1.2 (0.9–1.6)	1.0 (0.7–1.5)
	Secondary (9–12)	65 (23.0)	217 (77.0)	0.8 (0.6–1.2)	0.8 (0.5–1.1)
	Tertiary (college)	20 (20.0)	80 (80.0)	0.7 (0.42–1.2)	0.6 (0.3–1.1)
**Wealth index**	Lowest	165 (25.1)	492 (74.9)	1.0	1.0
	Second	65 (24.5)	200 (75.5)	0.9 (0.7–1.4)	0.7 (0.4–1.3)
	Middle	67 (24.5)	207 (75.5)	1.0 (0.8–1.3)	1.0 (0.5–2.2)
	Fourth	119 (29.1)	290 (70.9)	1.2 (0.9–1.6)	1.1 (0.5–2.5)
	Highest	114 (28.1)	291 (71.9)	1.2 (0.9–1.5)	1.2 (0.5–2.7)

**p < 0.05: significant association, *COR* Crude Odds Ratio, *AOR* Adjusted Odds Ratio

## Discussion

The study determined the prevalences of physical fights and physical attacks among adolescents in eastern Ethiopia. In addition, factors associated with these outcomes have been identified. Of those factors, being in-school and having ever chewed khat were associated with reduced likelihood to be physically attacked. For those in school, attending secondary or tertiary level of education had protective relationship with physical fighting. Sex was significantly associated with physical fight, with male adolescents two times more likely to engage in physical fights compared to female adolescents.

We reported that physical fights among adolescents is a public health concern in Ethiopia, with more than one-in-four (26.4%) of adolescents engaging in physical fight with their acquaintances. Likewise adolescents’ physical fights constitute a public problem in other countries, and our finding is relatively consistent with findings from studies conducted in Ghana (32%) [14], Egypt (31%) [15], Venezuela (31.2%) [16], Malaysia (27.4%) [17] and the Caribbean (28.6%) [12]. However, we noted lower rates of physical fight among adolescents in eastern Ethiopia compared to studies conducted among adolescents in Canada (35.6%) [18], Turkey (41.2%) [19], Chile (40.7%) [20], Jordan (43.3%) [21], Oman (47.6%) [22], Philippines (50.0%) [23], and also a study conducted in six Western Pacific countries ranged from 35 to 63% [24]. The prevalence of physical fighting among adolescents was quite high in Zambia (78%) [25]. Regardless of the figures reported by studies, physical fights during adolescence continue to be a public health challenge in several countries.

Adolescents have also experienced different form of violence in the form of physical attacks. In our study, we found out that the prevalence of physical attacks was at 5.8%. This is less than a study conducted in Oman that reported 38.8% [22], and a study in Jordan that reported 26.8% [21]. These differences in the prevalence of physical attack and physical fight might be related to social, cultural, religious, norms and beliefs variation across countries. Nonetheless, our study has not addressed those possible factors (social, cultural, religious, norms and beliefs variation) comprehensively. We believe our findings can be a good entry point for further studies.

Gender differences were found to be significant with physical fights among adolescents. A higher risk for physical fight among male adolescents compared to females is consistent with the literature from several countries [9, 17, 20, 24, 26–29], and in a paper that examined this among eighty eight countries [30]. The probable reason for male adolescents being engaged in physical fighting more than females might due to the old-fashioned gender-norms. Male perpetrators, aggressive and masculine behavior are accepted by community members [31]. Likewise, male adolescents who had peers with fighting history were more likely to be engaged in fighting and violence [32]. Being male was one of the statistically significant risk factors for physical fights which may be linked up with elevated psychological distress, and substance use [33]. We can anticipate that socially constructed gender-norms will remain an issues for physical fight unless adolescents can be shaped differently at early age. Research evidence on this is very limited in low income countries, and future studies should examine in greater detail why male are more engaged in physical fighting.

Our study findings support the established fact that education is a preventive factor for physical fights and attacks. We noted protective relationships between being in-school and greater levels of educational attainment against physical attack and physical fighting. This is consistent with studies in six Western Pacific countries [24], and findings from eighty eight countries [30], which may be related to structured time at school that reduce the opportunity for violence, enhanced skills for resolution of disagreements, and greater oversight by teachers. In addition, our finding of increased physical fight among those who worked for food or drink may be related to social status among their peers. This finding is consistent with a study conducted by Shaikh and his colleagues (2020) that indicated that adolescents who had food deprivation had almost double the risk for physical fights. Adolescents with food deprivation were 1.75 times more likely be involved in physical fight than their counter parts [34].

We observed that adolescents who ever chewed khat were less likely to be involved in physical violence. This finding was unexpected as khat is a psychostimulant chewable green plant followed by many risk taking behaviours, such as alcohol drinking and smoking [17, 35–37]. Khat chewing is very common in the study area [38], with devastating consequences and common mental disorders [39–41], which may have associations with physical fighting. In addition, adolescents may use khat to promote concentration on academic [37, 39, 42] or religious studies or worshiping [37, 39, 43] per local customs and beliefs. Nevertheless, the association between khat chewing and physical fighting among adolescents is not adequately researched in Ethiopia, and other khat plant cultivating countries. This is one of the new findings that needs further studies to explore in future research.

Overall, physical attacks and fights have important consequences for adolescent psychosocial development, educational attainment, self-confidence, intelligence [44], health [37, 45–47] and physical disability [46]. While there remains a paucity of evidence in this area, epidemiological studies in different settings and a systematic review in sub-Saharan African indicates that there is increasing recognition of the impact of violence on adolescent health and wellbeing [47, 48]. Further research is needed to determine the prevalence and factors associated with violence among adolescents and to evaluate links between educational and social factors in various settings. Approaches that aim to increase school enrollment and retention and programs to decrease violence should be prioritized in eastern Ethiopia and other similar low resource settings.

The current study has several limitations. With a cross-sectional design, we are unable to demonstrate causal relationships between the risk factors and violence. In addition, self-reported data may be subjected to participant recall bias, and it is possible that the true prevalence of physical attacks, and fights may be higher than these findings. In addition, the relationships between physical fights and khat chewing needs to be examined using longitudinal study designs and more depth exploration using qualitative studies.

## Conclusions

Physical attacks and fighting are common experiences for adolescents in Ethiopia. School enrolment is one of the protective factors for adolescents to reduce the risk of engaging in physical fights and attacks. Being male and engaged in work for food and drinks have put the adolescents’ at risk of physical fights. Away from the established fact of the risky nature of psychostimulant utilization for physical fights and attacks, the green psychostimulant substance (called khat) that is widely grown in eastern Ethiopia has been shown as a protective factor for adolescents’ from physical fights and attacks. Further research is needed to rigorously assess the relationship of khat chewing and adolescents’ physical violence. Our findings suggest that research and policy for adolescent health in Ethiopia should focus on community-based programs that transform old-fashioned masculine norms, and encourage adolescents’ school enrolment and retention to prevent violent behaviour.

## Data Availability

The participants de-identified data used for current study will be available upon submitting reasonable request from the corresponding author (AS) in either SPSS or Stata format and as per the permission obtained from senior project principals (YD, YB, NA, and WWF).

## Abbreviations

ARISE: Africa Research Implementation Science and Education
AOR: Adjusted Odds Ratio
CI: Confidence Interval
HDSS: Health Demographic Surveillance System
PCA: Principal Component Analysis
WHO: World Health Organization
